# *DSP* c.6310delA p.(Thr2104Glnfs*12) associates with arrhythmogenic cardiomyopathy, increased trabeculation, curly hair, and palmoplantar keratoderma

**DOI:** 10.3389/fcvm.2023.1130903

**Published:** 2023-03-15

**Authors:** Krista Heliö, Eveliina Brandt, Satu Vaara, Sini Weckström, Liisa Harjama, Riina Kandolin, Johanna Järviö, Katariina Hannula-Jouppi, Tiina Heliö, Miia Holmström, Juha W. Koskenvuo

**Affiliations:** ^1^ Heart and Lung Center, ERN GUARD-Heart Center, University of Helsinki and Helsinki University Central Hospital, Helsinki, Finland; ^2^ Department of Dermatology and Allergology, ERN-Skin Center, University of Helsinki and Helsinki University Central Hospital, Helsinki, Finland; ^3^ Radiology, HUS Diagnostic Center, University of Helsinki and Helsinki University Central Hospital, Helsinki, Finland; ^4^ Folkhälsan Research Center, Helsinki, Finland and Research Programs Unit, Stem Cells and Metabolism Research Program, University of Helsinki, Helsinki, Finland; ^5^ Department of Clinical Physiology and Nuclear Medicine, Turku University Hospital, Turku, Finland

**Keywords:** *DSP*, cardiomyopathy, arrhythmogenic cardiomyopathy, palmoplantar keratoderma, myocarditis, CMR

## Abstract

**Background:**

Pathogenic variants in *DSP* associate with cardiac and cutaneous manifestations including arrhythmogenic right ventricular cardiomyopathy, dilated cardiomyopathy, curly or wavy hair, and palmoplantar keratoderma (PPK). Episodes of myocardial inflammation associated with *DSP* cardiomyopathy might be confused in clinical work with myocarditis of other etiologies such as viral. Cardiac magnetic resonance imaging (CMR) may help in differential diagnosis.

**Methods and results:**

This study comprised 49 Finnish patients: 34 participants from families with suspected *DSP* cardiomyopathy (9 index patients and 25 family members) and 15 patients with myocarditis. All 34 participants underwent genetic testing and cardiac evaluation, and 29 of them also underwent CMR. Participants with the *DSP* variant, numbering 22, were dermatologically examined. The 15 patients with myocarditis underwent CMR and were evaluated during their hospitalization.

A heterozygous truncating *DSP* c.6310delA p.(Thr2104Glnfs*12) variant was confirmed in 29 participants. Only participants with the *DSP* variant had pacemakers and life-threatening ventricular arrhythmias. Of the participants with the *DSP* variant, 24% fulfilled cardiomyopathy criteria, and the median age at diagnosis was 53. Upon CMR, myocardial edema was found to be more common in patients with myocarditis. Both groups had a substantial percentage of late gadolinium enhancement (LGE). A ring-like LGE and increased trabeculation were observed only in participants with the *DSP* variant. All the studied participants with the *DSP* variant had PPK and curly or wavy hair. Hyperkeratosis developed before the age of 20 in most patients.

**Conclusions:**

The *DSP* c.6310delA p.(Thr2104Glnfs*12) variant associates with curly hair, PPK, and arrhythmogenic cardiomyopathy with increased trabeculation. Cutaneous symptoms developing in childhood and adolescence might help recognize these patients at an earlier stage. CMR, together with dermatologic characteristics, may help in diagnosis.

## Introduction

Arrhythmogenic right ventricular cardiomyopathy (ARVC) has been primarily recognized in patients with histological changes in the right ventricle and a tendency toward life-threatening ventricular arrhythmias, especially in athletes and young people (1, 2). In recent years, it has been increasingly reported that in addition to the classic subtype of ARVC with isolated right ventricular (RV) involvement, the disease may also present with left-ventricular (LV) involvement (3). The proposed concept of arrhythmogenic cardiomyopathy (ACM) reflects a broader clinical spectrum of the disease (4). In ACM, LV involvement can also be equal to or exceed RV involvement (5). There is currently no precise definition for ACM, but typical features include fibrofatty replacement of the myocardium and ventricular arrhythmias (6). Diagnostic criteria are lacking for the biventricular and left-ventricular subtypes of the disease, which may limit its recognition. ACM is associated with pathogenic variants in desmosomal genes, and half of the patients with classical ARVC have a pathogenic variant in desmosomal genes (7).

Desmosomes are intercellular junctions found both in the heart and in the skin. Desmoplakin, encoded by the *DSP* gene, anchors intermediate filaments to desmosomal plaques (8). In addition to ACM, variants in *DSP* have been associated with dilated cardiomyopathy (DCM) and in rare cases with left-ventricular non-compaction cardiomyopathy (LVNC) (3, 9–11). In addition to the exclusively cardiac phenotypes, variants in *DSP* have been associated with rare cardiocutaneous phenotypes with autosomal recessive (AR) or dominant (AD) inheritance. The cutaneous phenotype related to *DSP* variants includes curly or wavy hair and palmoplantar keratoderma (PPK), the epidermal thickening of the palms and soles (12–14). Carvajal disease is a cardiocutaneous syndrome with AR or AD inheritance characterized by a combination of PPK, woolly hair, and severe LV cardiomyopathy (15). More recently, AD *DSP* variants have been associated with left-dominant ACM and different degrees of PPK and sometimes curly or woolly hair (13, 16, 17).

The phenotypic expression in patients with *DSP* variants is variable. It has been suggested that *DSP* cardiomyopathy could be considered a distinct form of cardiomyopathy (18). In addition to the cutaneous features, patients have been reported to have episodes of myocardial inflammation, sometimes provoked by exercise (18, 19). These inflammatory episodes might be confused with myocarditis of other etiologies, which might delay diagnosis (20, 21). Cardiac magnetic resonance imaging (CMR) may help in differential diagnosis (22).

In addition to measuring the volumetrics of the heart, CMR can be used to characterize myocardial tissue. T1 and T2 relaxation time mapping techniques and extracellular volume (ECV) quantification help evaluate intracellular myocyte disruption and changes in the extracellular matrix (23). Late gadolinium enhancement (LGE) sequences detect myocardial fibrosis, scars, and edema (24). T1 mapping sequences are sensitive when assessing myocardial edema in acute myocarditis (25).

We previously reported the cardiac phenotype caused by the *DSP* c.6310delA p.(Thr2104Glnfs*12) variant, but cutaneous involvement and CMR findings were not assessed systematically (26). We now describe the cardiocutaneous phenotype associated with this *DSP* variant and compare the CMR findings with those from patients with clinically suspected viral myocarditis.

## Methods

### Subjects

This study comprised 49 Finnish participants. Out of these, 34 participants belonged to families with suspected *DSP* cardiomyopathy, 9 of them being index patients with the *DSP* c.6310delA p.(Thr2104Glnfs*12) variant and 25 being family members. The nine families included in this study were unrelated. The index patient (II.1) of family VII was recruited from the Department of Dermatology, Helsinki University Hospital. The other index patients were recruited from the Heart and Lung Center, Helsinki University Hospital.

Participants with clinically diagnosed viral myocarditis amounted to 15. They were treated at the Helsinki University Hospital. The inclusion criteria were age ≥18 years and CMR performed at the Helsinki University Hospital because of clinically suspected viral myocarditis. The exclusion criteria were suspected mRNA COVID-19 vaccination–induced myocarditis/myocardial inflammation or a verified COVID-19 infection at the time of myocarditis.

All participants gave written informed consent. This study was approved by the ethical review committee of The Department of Medicine, University of Helsinki (HYKS 26/99, HYKS 16/99, HYKS 17/99, HYKS 19/2000, HYKS 8/2000, HUS/3225/2018 Dnro 307/13/January 03, 2011, TMK11§274, December 16, 2015, HUS/2004/2018, and HUS/2675/2019). Statistics Finland (Dnro TK-53-345-17), Finnish Institute for Health and Welfare, and the Ministry of Social Affairs and Health gave permission to obtain clinical data from deceased patients for research purposes (THL/1078/5.05.00/2020). This study complied with the Declaration of Helsinki.

### Molecular genetic studies

The DNA samples of 9 index patients and 25 family members were studied. Patients with myocarditis did not undergo genetic testing. The Blueprint Genetics laboratory (Espoo, Finland) carried out the genetic testing.

All index patients were referred to genetic testing by their physicians as part of their diagnostic evaluation. In families I, V, VI, VII, and VIII, the index patient was tested using a gene panel. Bidirectional Sanger sequencing revealed the presence of the *DSP* c.6310delA p.(Thr2104Glnfs*12) variant in other participants. In families II, III, IV, and IX, a family member of the index patient underwent genetic panel testing that recognized the *DSP* c.6310delA p.(Thr2104Glnfs*12) variant in the family. However, they were not considered index patients of this study as they were either deceased or otherwise could not be evaluated as part of this study.

The palmoplantar keratoderma panel including 26 genes associated with PPK was used in the index patient of Family VII. For three participants, the *DSP* variant was revealed using an NGS panel covering 101 genes associated with cardiomyopathies. Other NGS panels covering 27, 86, 93, 177, and 217 genes associated with cardiomyopathies were each used in one participant. Mitochondrial DNA was analyzed as part of the panel testing in five participants.

Variant nomenclature was based on GenBank accession NM_004415.2 (*DSP*), with the nucleotide one being the first nucleotide of the translation initiation codon ATG. The classification scheme of the American College of Medical Genetics and Genomics (ACMG) was used to determine the pathogenicity of this *DSP* variant (27).

### Cardiac evaluation

All 34 participants from the families with suspected *DSP* cardiomyopathy underwent cardiac evaluation with regard to patient history, family history, 12-lead electrocardiogram (ECG), echocardiography, physical examination, and appropriate laboratory tests. The laboratory tests included TnI, CRP, proBNP, lactate, CK-MbM, and inflammatory tests (IL-2R, IL-6, IL-8, TNF, ECP, and hs-CRP). Of these, 29 participants underwent CMR. Some participants also underwent a stress test or Holter monitoring. The 15 patients with myocarditis underwent CMR and were evaluated by their physicians during their stay at the hospital. Clinical data, including previous cardiac evaluation, were obtained from all available hospital records.

DCM diagnostic criteria included systolic dysfunction [LV ejection fraction (LVEF) < 45%] and dilatation of the LV [left-ventricular end-diastolic diameter (LVEDD) > 27 mm/m^2^]. Patients diagnosed with DCM had neither severe coronary artery disease nor abnormal loading conditions such as valvular disease or hypertension. Patients with ARVC were diagnosed originally by their physicians using the existing criteria available at that time. The diagnosis was now verified using the 2010 ESC Task Force Criteria (1).

Participants were considered affected if they had at least one of the following: unexplained elevated Troponin I (TnI) concentration (>45 ng/L), atrial fibrillation (AF), or flutter (AFL) at under 50 years, a significant amount of ventricular extrasystoles (VES) or supraventricular extrasystoles (SVES) in Holter monitoring (>500/24 h), conduction defects (left bundle branch block, right bundle branch block, or atrioventricular block of any degree), LV dilatation (≥117% of the predicted LVEDD using Henry's formula), systolic dysfunction, or an unexplained abnormal CMR (28, 29). Unexplained abnormal CMR included increased trabeculation, abnormal LV or RV size, or abnormal LGE distribution not explained by any other factors such as myocarditis or athlete's heart. Trabeculation was assessed using the following criteria: a ratio of non-compacted to compacted (NC/C) myocardium diameter of 2–2.3 in the end diastole was considered mildly prominent and LVNC criteria fulfilling with an NC/C ratio of >2.3 in the end diastole.

### CMR technique

CMR was performed with 1.5 T MR (Avantofit; Siemens, Erlangen, Germany) using a 32-channel receiver cardiac coil. Breath-hold cine MR was performed using retrospectively electrocardiographically gated segmented true fast imaging with a balanced steady-state free precession (bSSFP) sequence. To assess left and right ventricular volumes and ejection fractions, cine CMR images were obtained in vertical and horizontal long axes and a stack of short-axis planes covering both ventricles. Quantitative myocardial T2 mapping was performed in a breath-hold fashion by using a T2-prepared bSSFP sequence to analyze myocardial edema. Myocardial edema was assessed using T2 weighted short Tau inversion recovery (STIR) imaging. For myocardial T1 mapping and extracellular volume quantification, three (apex, mid-ventricular, basis) short-axis planes and a four-chamber long-axis plane were acquired before and after contrast injection using the shortened modified look-locker inversion recovery (ShMOLLI) sequence. Ten minutes after injection of a contrast agent (gadoterate meglumine, Dotarem® 0.2 mmol/kg), LGE images were acquired in the same views as for cine images, using inversion recovery spoiled gradient echo (IR-SPGR) and phase-sensitive inversion recovery (PSIR) sequences.

### CMR image analysis

CMR image analysis was performed using QMass MR software® (version 7.6, Medis Medical Imaging Systems, Leiden, Netherlands). Measurements of left and right ventricular volumes and ejection fraction were segmented semiautomatically by tracking the endocardial borders of both ventricles with a segmentation tool developed for this purpose. For myocardial tissue characterization (myocardial edema and fibrosis), T2, precontrast (native) T1, and postcontrast T1 relaxation times were calculated and extracellular volume fraction quantified. Myocardial edema was first determined by using T2 STIR images. The T2 signal intensity (SI) ratio was calculated by dividing the SI of the myocardium by the SI of the skeletal muscle in the same short-axis slice. The location, type, and extent of LGE was determined visually by using the AHA 17 segment model of the left ventricle. Then, epicardial and endocardial LV contours were placed semiautomatically on all LGE SA images and LGE mass and percentage was quantified by using the full width at half maximum (FWHM) method. In addition to segmental analysis, the localization and extent of LGE was assessed by using visual impression. LGE was considered circular when appearing to cover the whole myocardium.

### Dermatologic evaluation

Eight of the nine index patients underwent a detailed clinical dermatologic examination performed by at least one dermatologist. In addition, the index patient of family I was interviewed *via* telephone and evaluation was complemented by photographs because in-person evaluation was not possible. A systematic dermatologic examination of 13 family members with the *DSP* variant was performed: 10 from family I, both family members in family II, and the only family member in family IV. The clinical examination was supplemented with light microscopy of hair samples when available.

### Statistical analysis

A comparison of groups was performed by using Fisher's exact test for categorical variables and the Mann–Whitney-U test for continuous variables. *P*-values <0.05 were considered statistically significant.

## Results

### Genetic studies

**Figure 1:**
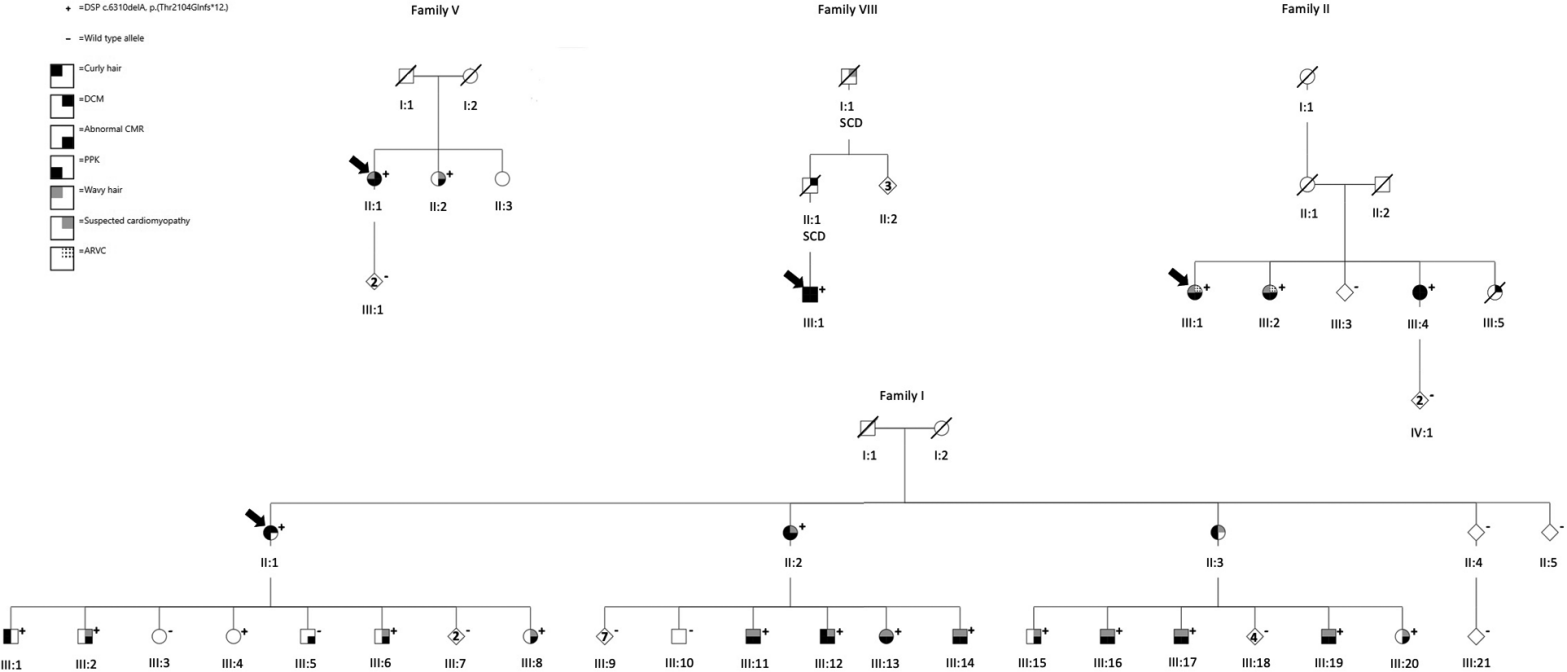
Pedigrees of families I, II, V, and VIII. The arrows indicate the index patients. Genotype: + heterozygous for the *DSP* p.(Thr2104Glnfs*12),—wild-type allele. *Right upper quadrant*: black denotes individuals with DCM, the dotted lines denote ARVC, and gray represents suspected cardiomyopathy. *Right lower quadrant*: black denotes abnormal CMR. *Left upper quadrant*: black indicates curly hair, and gray indicates wavy hair. *Left lower quadrant*: black represents verified PPK. SCD is short for sudden cardiac death. DCM, dilated cardiomyopathy; CMR, cardiac magnetic resonance imaging; ARVC, arrhythmogenic right ventricular cardiomyopathy; PPK, palmoplantar keratoderma.

We identified a heterozygous *DSP* c.6310delA p.(Thr2104Glnfs*12) variant in 29 patients in nine families (Figure 1, Supplementary Figure S1). The variant causes a frameshift, which leads to a premature stop codon at residue 12 in a new reading frame. The variant affects both *DSP* transcripts and is predicted to cause loss of protein function through nonsense-mediated mRNA decay from the other allele or protein truncation (2104 out of 2871 amino acids).

According to gnomAD (accessed on May 2022), there are 13 individuals heterozygous for the *DSP* c.6310delA, p.(Thr2104Glnfs*12) variant, of which 11 are of Finnish ethnicity. The total allele frequency in the Finnish population is 0.0005081, as opposed to a total allele frequency of 0.00005170.

### Index patients

#### Family I

The index patient (II.1) was a 69-year-old woman with no known family history of cardiomyopathy. She had been previously healthy, but at 52 years, she experienced sudden VF at home with successful resuscitation. Afterward, she was diagnosed with DCM and an ICD was implanted, which was later updated to a CRT-D. Five years after the implantation, she experienced two episodes of VF. Echocardiography showed that the LVEDD was 70 mm, and LVEF was 25%, whereas RV size and function were normal. Her proBNP levels varied between 993 and 1464 ng/L during the past few years (reference range <352 ng/L for women over 65). Her TnI level was the highest at 993 ng/L (reference range <45 ng/L).

She reported to have dry skin on the heels. The photographs showed a mild plantar hyperkeratosis of the first metatarsals and the heels and slightly prominent plantar creases. In childhood, she had eczema on the ankles and popliteal fossae and fissuring of the plantar skin of the first toes. She had no palmoplantar hyperhidrosis, pain, or aquagenic whitening, i.e., after water exposure, the hyperkeratosis turns white and spongy. Her hair was curly with good growth.

#### Family II

The index patient (III.1) was a 57-year-old woman diagnosed with ARVC and a family history of ARVC/biventricular ACM. The patient and her family members (III.2) fulfilled the 2010 Task Force Criteria (1). Echocardiography showed that the LVEDD was 50 mm and LVEF 35%. Upon CMR, microaneurysms were observable in the RV. In Holter monitoring, frequent VES and NSVT were observed, and she received an ICD. Her maximum TnI level was 47 ng/L, and the proBNP level was 510 ng/L (reference range <222 ng/L).

She had focal plantar hyperkeratosis in both heels, both I and V toes, right II–IV toes, and the left IV toe ever since she attained adulthood. The areas of hyperkeratosis had aquagenic whitening and occasional painful fissuring and itching, but she had no hyperhidrosis. The palmar skin of her fingers was noted to be scaly and dry, and her palmar creases were slightly prominent. Her hair was wavy and brittle. Hair microscopy showed slightly narrowed regions along the hair shafts.

#### Family III

The index patient (III.3) was a 44-year-old man with a family history of DCM. In recent years, he had one episode of suspected myocarditis. His TnI level was 16,105 ng/L, and CRP was 4 mg/L, and upon CMR, he had a ring-like LGE. The findings were deemed compatible with an inflammatory process. Afterward, he suffered from multiple episodes of chest pain with slightly elevated TnI levels (28–113 ng/L) and simultaneously a normal proBNP of 50 ng/L (reference range <84 ng/L). Echocardiography revealed that the LVEDD was 49 mm and LVEF 62%. He had no recorded arrhythmias.

From the age of 17, he had hyperkeratosis of the heels and first toes. He complained of occasional pain related to fissuring, aquagenic whitening of the hyperkeratosis, and slight hyperhidrosis of the feet. In childhood and adolescence, he had wavy and well-growing hair, but from the age of 30 years onward, he had male pattern baldness (androgenic alopecia). His feet showed hyperkeratosis of the heels as well as medial and dorsal aspects of the first toes. His I–III fingertips were rough and dry. His palmar and plantar creases appeared to be slightly prominent. Hair microscopy showed no hair shaft alterations.

#### Family IV

The index patient (II.2) was an asymptomatic 60-year-old man with a family history of DCM. Echocardiography showed that the LV was dilated (LVEDD 60 mm, 120% of the predicted LVEDD using Henry's formula) with normal systolic function. LV size had since reduced, and the latest LVEDD was 54 mm and LVEF was 66%. His TnI was 4 ng/L and proBNP was 265 ng/L (reference range <194 ng/L). He had no recorded arrhythmias.

He had focal hyperkeratosis of the heels, foot pads, and toes from the age of 20 as well as a single hyperkeratotic lesion on the palmar aspect of the left second finger. He had plantar hyperhidrosis as well as aquagenic whitening and occasional pain of the hyperkeratotic areas. Toenails had been hypertrophic since age 20 years. Palmoplantar creases were slightly prominent. In addition, he had suffered from atopic eczema since infancy. He had wavy hair in childhood and male pattern baldness in adulthood.

#### Family V

The index patient (II.1) was a 63-year-old woman with a family history of DCM. At 61, she developed shortness of breath during exercise and was diagnosed with DCM. Echocardiography showed that the LVEDD was 54 mm and LVEF 45%. CMR demonstrated a normal RV function, dilated LV, and inferolateral LGE. She suffered from palpitations, but Holter monitoring revealed VES <500/24 h. Her TnI levels were slightly elevated over the years (46–66 ng/L), and proBNP levels were the highest at 672 ng/L (reference range <222).

Her right I–III fingertips were slightly rough, and her plantar creases were slightly prominent. She exhibited an isolated mild focal keratoderma of both heels and the medial aspects of the first toes along with wavy hair. She described her palmoplantar skin to be symptomless and her hair wavy with normal growth.

#### Family VI

The index patient (II.1) was a 59-year-old woman who was diagnosed with DCM at 43. She suffered an ischemic stroke at 49, and the etiology was deemed cardiogenic. She suffered from paroxysmal AF, NSVT, and VES. Echocardiography showed that the LVEDD was 57 mm and LVEF 35%. CMR demonstrated a normal RV, dilated LV, and inferolateral subepicardial LGE. TnI levels were normal, and proBNP reached a maximum of 391 ng/L (reference range <222).

She had a slightly progressive hyperkeratosis of the heels since the age of 20 years and of the dorsal aspect of the first toes. Her palmar skin was scaly and dry along with palmar eczema. The index patient had atopic eczema since childhood, but her hands were affected in recent years. There was aquagenic whitening of the hyperkeratosis and occasional painful fissuring but no hyperhidrosis. Her hair was curly and brittle.

#### Family VII

The index patient (II.1) was a 41-year-old man. He suffered from palpitations and occasional chest pain. Echocardiography revealed an LVEDD of 45 mm and an LVEF of 67%. CMR demonstrated a normal LV and RV function and a slight subepicardial/intramyocardial LGE. His TnI was 4 ng/L and proBNP 72 ng/L (reference range <84 ng/L).

He was initially recruited from the Department of Dermatology, Helsinki University Hospital, where during a dysplastic nevus removal he was diagnosed with focal PPK. He had focal PPK on the heels and first toes since the age of 15 and on the palms since the age of 30. He reported occasional painful fissuring and aquagenic whitening of the hyperkeratosis, localized hyperhidrosis of the feet, and milder generalized hyperhidrosis. He also had a slight keratosis pilaris of the upper arms. His hair was curly and well growing. Hair microscopy showed a normal hair shaft structure.

#### Family VIII

The index patient (III.1) was a previously healthy 30-year-old man with a paternal family history of arrhythmias and sudden cardiac death (SCD). His father suffered from arrhythmias and died suddenly at the age of 37. His paternal grandfather had a dilated LV at the age of 34 and died suddenly at the age of 57. The index patient had an episode of chest pain with a TnI level of 7,450 ng/L and was admitted to hospital with suspected myocarditis. On the next day in hospital, his TnI level rose to 72,422 ng/L and he suffered from NSVT. Later that day, he experienced VT, which degenerated to VF, but he was successfully resuscitated. CMR demonstrated LV dysfunction and a ring-like LGE and an extensive LGE in the LV. His proBNP increased to 3969 ng/L during hospitalization, but it later reduced to 164 ng/L (reference range <84 ng/L). He received an ICD.

He had a slight palmoplantar hyperkeratosis of the right II–III fingers, heels, first toes, and right V toe. He had a slight plantar hyperhidrosis but otherwise felt his skin to be symptomless. His hair was curly with normal growth, and hair microscopy did not show any hair shaft abnormalities.

#### Family IX

The index patient (II.1) was a 30-year-old man with a family history of DCM/ARVC. He had suffered from multiple episodes of myocarditis in his twenties. In recent years, he has had 8,000–13,000 VES upon a 24 h Holter monitoring. Echocardiography revealed that the LVEDD was 55mm and LVEF 54%. CMR demonstrated a dilated LV and subepicardial LGE. The TnI level was a maximum of 86 ng/L, and proBNP was 50 ng/L (reference range <84 ng/L).

He had a mild focal palmar hyperkeratosis of the II–IV metacarpophalangeal joints and focal plantar hyperkeratosis of the first toes and heels since childhood. Aquagenic whitening of the plantar lesions, palmoplantar hyperhidrosis, as well as light generalized hyperhidrosis, were reported. His hair was curly and thick, but hair microscopy showed no structural abnormalities.

### Cardiac evaluation

Altogether, 34 patients from 9 families (9 index patients and 25 family members) with suspected hereditary *DSP* cardiomyopathy underwent cardiac evaluation (Table 1). The nine index patients and 20 of the 25 family members were heterozygous for the *DSP* variant. Clinical data from hospital records were evaluated for 15 patients with myocarditis.

**Table 1:** Cardiac evaluation of patients with myocarditis, participants carrying the *DSP* variant, and wild-type family members.

	Myocarditis	*DSP*+	*DSP*+	*DSP*−
		index patient	family member	family member
	*n* = 15	*n* = 9	*n* = 20	*n* = 5
Age	28 (22–33)	50 (40–57)	38 (29–52)	37 (34–49)
Males	13 (87)	5 (56)	11 (55)	3 (60)
CMR performed^a^	15 (100)	7 (78)	19 (95)	3 (60)
Echocardiography	14 (93)	9 (100)	19 (95)	5 (100)
LVEDD (mm)	51 (47–56)	54 (50–56)	53 (51–55)	53 (50–54)
LVEDD (%)	105 (100–108)	109 (108–116)	108 (105–117)	106 (105–110)
LVEF (%)	61 (55–64)	50 (35–62)	57 (54–63)	61 (52–64)
**Arrhythmias**				
VF	0	2 (22)	0	0
VT	0	1 (11)	0	0
NSVT	2 (13)	4 (44)	3 (15)	1 (20)
VES	1 (7)	3 (33)	4 (20)	2 (40)
SVES	0	0	2 (10)	0
AFL	0	0	1 (5)	1 (20)
AF	1 (7)	1 (11)	1 (5)	0
ICD	0	2 (22)	3 (15)	0
CRT-D	0	1 (11)	0	0
**Laboratory test**				
CRP (mg/L)	19 (4–31)	3 (3–7)	3 (0)	3 (2–3)
TnI (ng/L)	6443 (343–16,610)	15 (7–47)	3 (3–10)	3 (0)

*DSP* +, heterozygous for the *DSP* variant; *DSP* –, wild-type allele; LVEDD, left-ventricular end-diastolic diameter; LVEDD (%), LVEDD compared with predicted LVEDD using Henry's formula; LVEF, left-ventricular ejection fraction; VF, ventricular fibrillation; VT, ventricular tachycardia; NSVT, non-sustained ventricular tachycardia; VES, ventricular extrasystoles (>500/24 h); SVES, supraventricular extrasystoles (>500/24 h); AFL, atrial flutter; AF, atrial fibrillation; ICD, implantable cardioverter-defibrillator; CRT-D, cardiac resynchronization therapy defibrillator; CRP, C-reactive protein; TnI, troponin I.

For continuous variables, numbers are presented as median (interquartile range) and for categorical variables as number (percentage).

^a^ Detailed findings in Tables 2,3.

The median age for index patients and relatives carrying the *DSP* variant was higher than that of patients with myocarditis (50 vs. 28 years, *P* = 0.002 and 38 vs. 28 years, *P* = 0.03, respectively). The majority of patients with myocarditis were males.

Echocardiography was conducted on all 29 patients with the *DSP* variant (9 index patients and 20 family members), 5 family members without the *DSP* variant, and 14 patients with myocarditis. LVEF seemed to be slightly lower in index patients than in the other three groups, but the difference was not statistically significant. The LVEDD, when compared with predicted LVEDD using Henry's formula, tended to be slightly larger in index patients and family members with the *DSP* variant, although the differences between the four groups were not clinically or statistically significant.

One family member with and one without the *DSP* variant had AFL at under 50 years. Paroxysmal AF was observed in one index patient at over 50 years, at under 50 years in one family member with the *DSP* variant, and in one patient with myocarditis. NSVT was observed in 4/9 index patients, 3/20 family members with the *DSP* variant, 1/5 family member without the *DSP* variant, and 2/15 myocarditis patients. Only the index patients had sustained VT or VF. Only participants with the *DSP* variant had pacemakers: two index patients and three family members had an ICD and one index patient a CRT-D.

Inflammatory markers were available from 23/29 participants with the *DSP* variant and 4/5 relatives without the *DSP* variant. For those carrying the *DSP* variant, all laboratory tests, including TnI and CRP, were performed in a stable phase as part of this study, and for patients with myocarditis, the test were conducted during the period of hospitalization. ECP was slightly above the reference range (<16 µg/L) in 3/4 family members without the *DSP* variant (21–25 µg/L) and in 3/20 family members with the *DSP* variant (21–41 µg/L) but in none of the index patients. IL-8 was above the reference range (<15 pg/L) in one index patient (29 pg/L). The participants did not demonstrate values outside of the reference range in the other inflammatory markers. Laboratory values for CRP and TnI were available from almost all participants with the *DSP* variant (21/29 and 28/29, respectively) and from all 15 patients with myocarditis. CRP and TnI levels were higher in patients with myocarditis than in index patients (19 vs. 3 mg/L, *P* = 0.07 and 6,443 vs. 15 ng/L, *P* < 0.001, respectively) and family members with the *DSP* variant (19 vs. 3 mg/L, *P* < 0.001 and 6,443 vs. 3 ng/L, *P* < 0.001, respectively).

Of the index patients, 4/9 were considered affected, 4/9 fulfilled DCM diagnostic criteria, and 1/9 fulfilled ARVC diagnostic criteria but had biventricular disease. Of the family members with the *DSP* variant, 1/20 fulfilled DCM diagnostic criteria and 1/20 fulfilled ARVC diagnostic criteria but had biventricular disease. In addition, 16/20 had abnormal findings upon cardiac evaluation and were considered affected. Of the family members with the *DSP* variant, 2/20 had no abnormal findings and were considered not affected. The median age at cardiomyopathy diagnosis was 53. Of the five family members without the *DSP* variant, 1/5 had no abnormalities, 1/5 had athlete's heart, 1/5 had AFL at age <50 years, and 2/5 had frequent VES (>500/24 h). Of the patients with myocarditis, none fulfilled any cardiomyopathy criteria.

### Cardiac magnetic resonance imaging

CMR analysis included 26 participants with the *DSP* variant and 15 patients with myocarditis. Patients with myocarditis and participants with the *DSP* variant presented with similar findings in CMR volumetric analyses (Table 2). There was no difference between the groups in terms of the global mean T1 or T2 relaxation time. Trabeculation, however, was markedly more common in participants with the *DSP* variant (0 vs. 50%, *P* = 0.001); six of the participants with the *DSP* variant had LVNC criteria fulfilling trabeculation (a ratio of NC/C myocardium >2.3 in the end diastole) and seven had milder trabeculation (a ratio of NC/C myocardium of 2–2.3 in the end diastole). Both groups demonstrated equal amounts of pericardial effusion and abnormalities in wall motion and regional T1 mapping. Myocardial edema was more often associated with myocarditis than with *DSP* variant (73.3% vs. 19.2%, *P* < 0.001). Three family members with the *DSP* variant showed no abnormalities upon CMR.

**Table 2:** Comparison of CMR findings between patients with myocarditis and participants with the *DSP* variant.

	Myocarditis	*DSP*+	*p*-value
	*n* = 15	*n* = 26	
LV EF (%)	56 (54–60)	54 (46–58)	0.086
LV EDV (mL/m^2^)	101 (84–109)	100 (92–111)	0.799
LV ESV (mL/m^2^)	43 (35–49)	49 (41–54)	0.121
LV SV (mL/m^2^)	58 (48–61)	51 (44–61)	0.289
LA (m^2^)	25 (24–27)	25 (21–26)	0.242
RV EF (%)	57 (52–60)	57 (52–62)	0.779
RV EDV (mL/m^2^)	97 (92–111)	91 (83–101)	0.096
RV ESV (mL/m^2^)	45 (39–48)	41 (34–47)	0.253
RV SV (mL/m^2^)	54 (48–61)	51 (44–60)	0.242
RA (m^2^)	24 (22–26)	22 (20–24)	0.067
Global mean T1 relaxation time	998 (967–1,046)	997 (980–1,015)	0.841
Global mean T2 relaxation time	47 (43–48)	48 (47–50)	0.108
Global mean ECV	—	28 (24–31)	
Trabeculation	0	13 (50.0)	0.001
LVNC criteria fulfilling trabeculation	0	6 (23.1)	0.070
Mildly prominent trabeculation	0	7 (26.9)	0.035
Wall motion abnormality	3 (20.0)	9 (34.6)	0.480
Regional T1 mapping abnormality	11 (73.3)	16 (66.7)	0.734
Myocardial edema	11 (73.3)	5 (19.2)	<0.001
Pericardial effusion	3 (20.0)	2 (7.7)	0.336
Pleural effusion	0	0	
Normal CMR	0	3 (11.5)	0.287

*DSP* +, heterozygous for the *DSP* variant; LV, left ventricle; EF, ejection fraction; EDV, end-diastolic volume; ESV, end-systolic volume; SV, stroke volume; LA, left atrium; RV, right ventricle; RA, right atrium; ECV, extracellular volume; LVNC, left-ventricular non-compaction cardiomyopathy.

Numbers are presented as median (interquartile range) for continuous variables and number (percentage) for categorical variables. *P*-values for continuous variables were calculated by using the Mann–Whitney-U test and for categorical values with Fisher's exact test.

**Figure 2:**
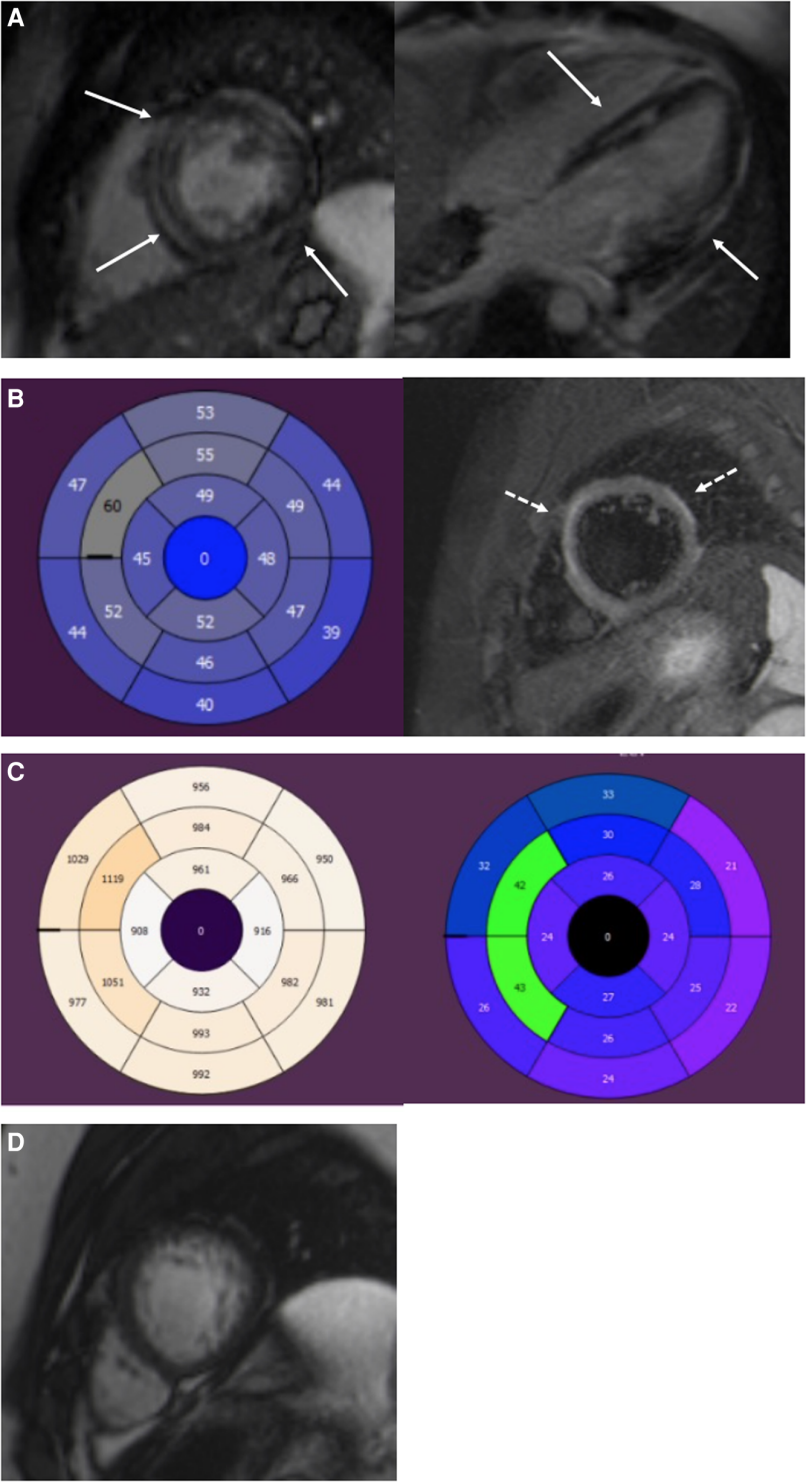
A 19-year-old male with *DSP* cardiomyopathy. (**A**) Short-axis and four-chamber LGE images show a midmyocardial and subepicardial ring-like enhancement of the LV (arrows). (**B**) AHA 17 segment Bull's eye of the LV. T2-mapping shows elevated T2 relaxation times (>52 ms) in the anteroseptal and anterior segments referring to myocardial edema. T2 fs image with myocardial edema in the anterior septum and anterolateral wall (dotted arrows). Edema appears as a high signal intensity. (**C**) T1 mapping bulls eye figure with high T1 relaxation times (>1,000 ms) in the anteroseptal segments of the LV. ECV is expanded in the same segments (>30%). (**D**) Short-axis cine image of the LV apex. Increased trabeculation of the apex but not fulfilling the LVNC criteria (>2.3). The ratio of the end-diastolic thickness of the non-compacted and compacted myocardial layers was 2.2. LGE, late gadolinium enhancement; LV, left ventricular; ECV, extracellular volume; LVNC, left-ventricular non-compaction cardiomyopathy.

**Figure 3:**
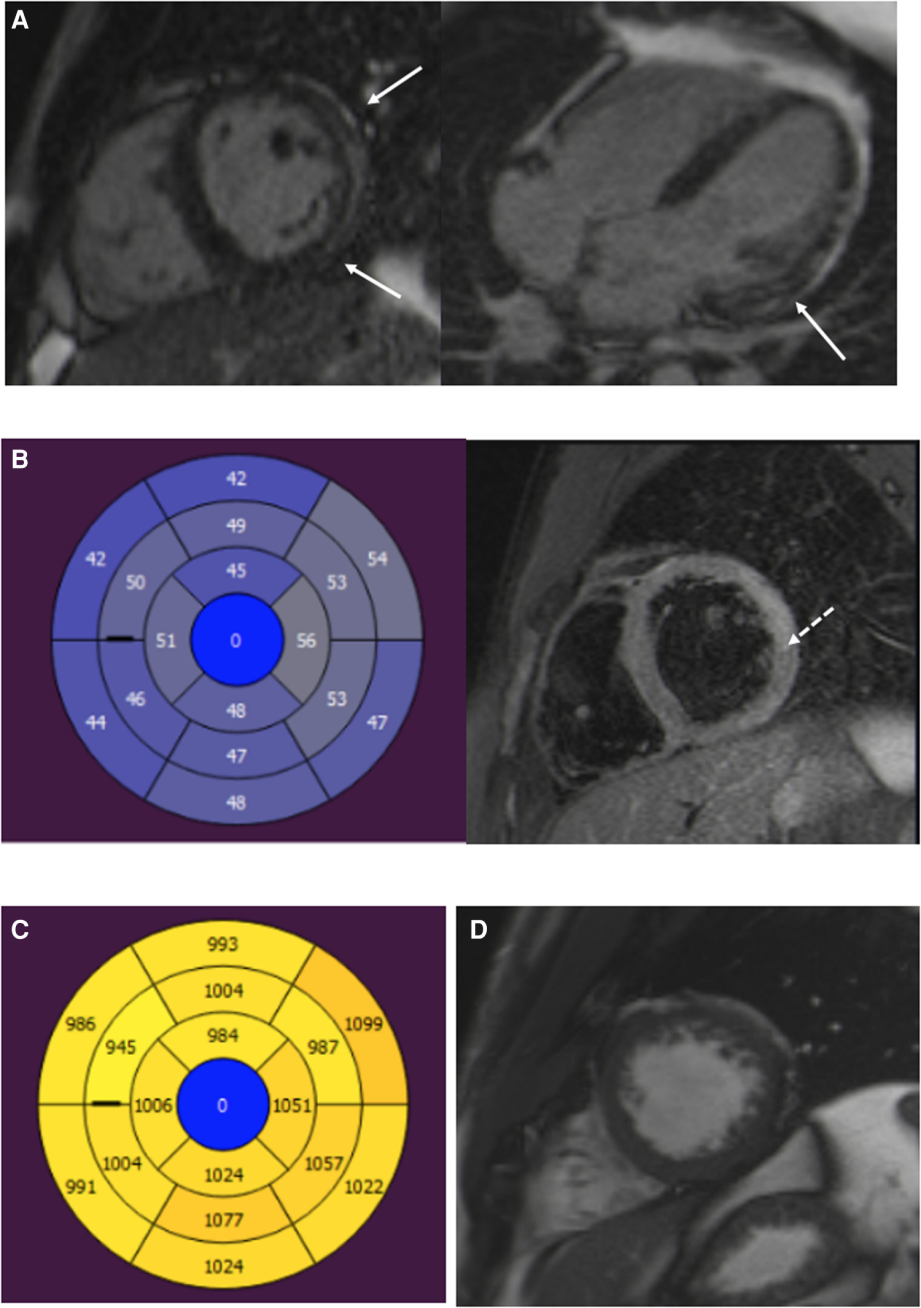
A 22-year-old male patient with myocarditis. (**A**) Short-axis and four-chamber LGE images show subepicardial enhancement of the lateral wall (arrows). (**B**) T2 mapping and T2 fs images show myocardial edema in the lateral wall (dotted arrow). (**C**) T1 relaxation times are elevated in the inferior and lateral segments. (**D**) Normal trabeculation of the LV apex. LGE, late gadolinium enhancement; LV, left ventricular.

**Figure 4:**
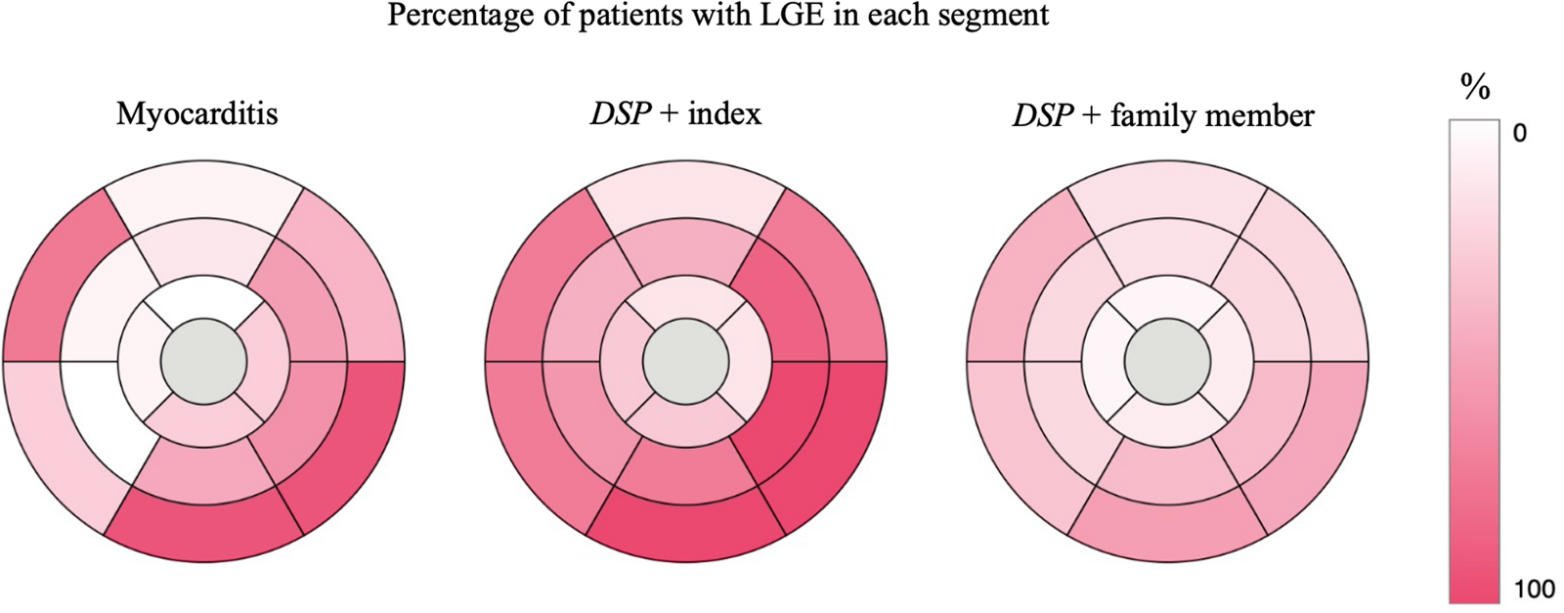
AHA 17 segment Bull's eye of the LV showing the percentage of participants with LGE in each segment. *DSP* +, heterozygous for the *DSP* variant. LGE, late gadolinium enhancement; LV, left ventricular.

Index patients and patients with myocarditis had a substantial percentage of LGE (Table 3, Figures 2–4). Eight family members with the *DSP* variant presented with no LGE, whereas all index patients and all patients with myocarditis presented with LGE. The LGEs were mostly located in the basal inferior/inferolateral and in the mid-inferior/inferolateral segments and their neighboring segments. In participants with the *DSP* variant, LGEs were also frequent in these segments, but the LGE distribution was slightly more even. A circular LGE was observed only in participants carrying the *DSP* variant.

**Table 3:** Distribution of LGEs in patients with myocarditis and in participants with the *DSP* variant.

		Myocarditis	*DSP*+ index patient	*DSP*+ family member
		*n* = 15	*n* = 7	*n* = 19
LGE %		14 (8–16)	14.5 (11–17)	0 (0–14)
**Segment with LGE**				
1 Basal anterior		1 (6.7)	1 (14.3)	3 (15.8)
2 Basal anteroseptal		11 (73.3)	5 (71.4)	8 (42.1)
3 Basal inferoseptal		4 (26.7)	5 (71.4)	6 (31.6)
4 Basal inferior		14 (93.3)	7 (100)	10 (52.6)
5 Basal inferolateral		14 (93.3)	7 (100)	9 (47.4)
6 Basal anterolateral		6 (40.0)	5 (71.4)	4 (21.1)
7 Mid-anterior		2 (13.3)	3 (42.9)	3 (15.8)
8 Mid-anteroseptal		1 (6.7)	3 (42.9)	4 (21.1)
9 Mid-inferoseptal		0	4 (57.1)	4 (21.1)
10 Mid-inferior		7 (46.7)	5 (71.4)	7 (36.8)
11 Mid-inferolateral		9 (60.0)	7 (100)	7 (36.8)
12 Mid-anterolateral		8 (53.3)	6 (85.7)	4 (21.1)
13 Apical anterior		0	1 (14.3)	1 (5.3)
14 Apical septal		1 (6.7)	2 (28.6)	1 (5.3)
15 Apical inferior		4 (26.7)	2 (28.6)	2 (10.5)
16 Apical lateral		4 (26.7)	1 (14.3)	2 (10.5)
17 Apex		0	0	0
Circular LGE		0	2 (28.6)	4 (21.1)
No LGE		0	0	8 (42.1)

*DSP* +, heterozygous for the *DSP* variant, LGE, late gadolinium enhancement.

Distribution of LGE in the left ventricle by the AHA 17 segment model. Numbers are presented as median (interquartile range) for continuous variables and number (percentage) for categorical variables.

### Dermatologic evaluation

**Figure 5:**
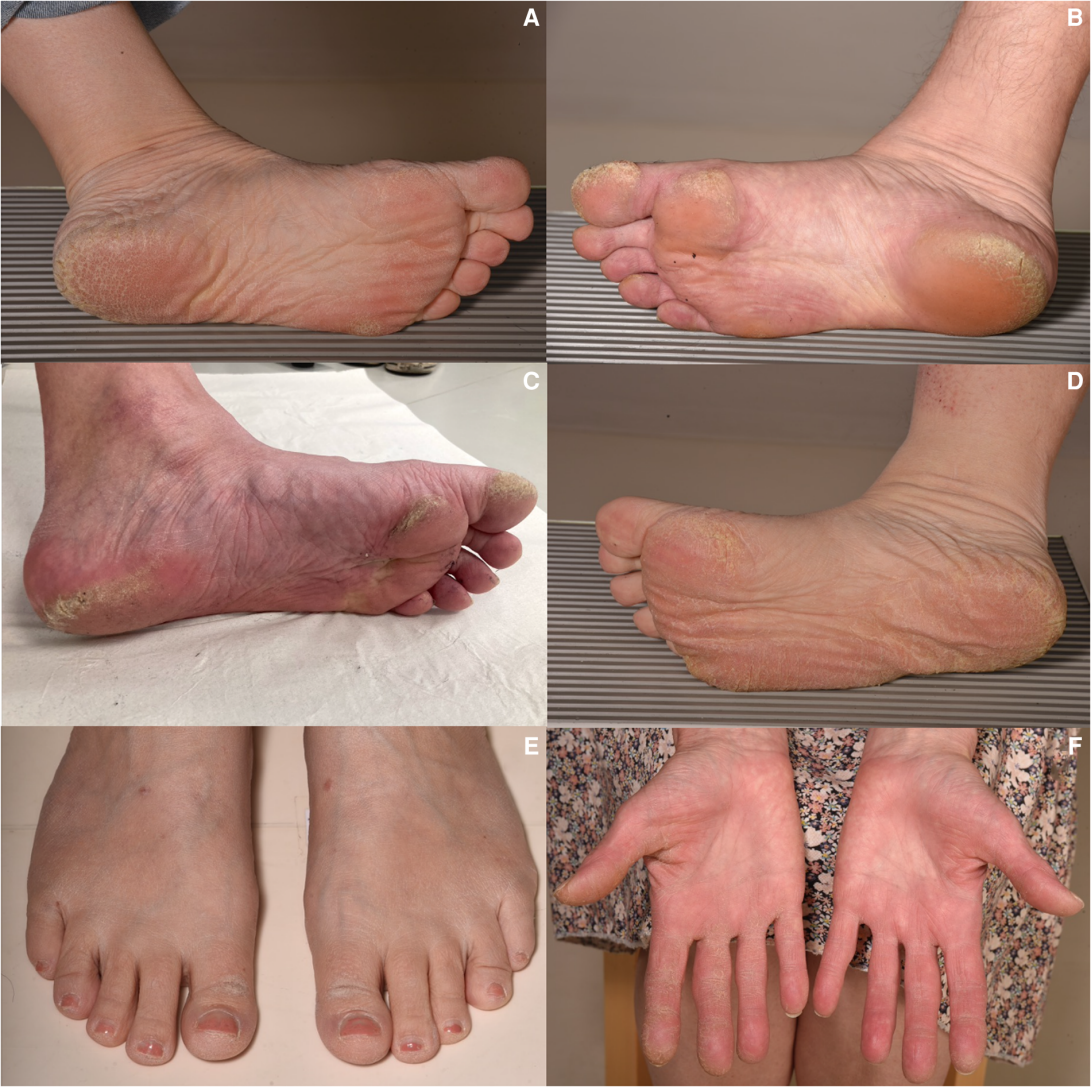
Clinical characteristic features of the palmoplantar skin in participants with the *DSP* c.6310delA p.(Thr2104Glnfs*12) variant. Note the different degrees of focal hyperkeratosis focusing on the first toes, footpad, and heels (**A–C**). One participant, a 40-year-old man, exhibited diffuse PPK (**D**) and two index patients had mild hyperkeratosis also on the dorsal aspect of the first toes in addition to focal plantar hyperkeratosis (**E**). Some of the participants also showed palmar hyperkeratosis mainly on the fingers and/or fingertips (**F**). PPK, palmoplantar keratoderma.

Altogether, 22 patients with the *DSP* variant (9 index patients and 13 family members from three families) underwent dermatologic evaluation. The dermatologic phenotype was similar in the index patients and family members, although PPK varied from slight to severe (Figure 5, Table 4, Supplementary Table S1). All studied patients exhibited PPK, most commonly focal PPK (21/22), and of the participants one had diffuse PPK (1/22). The reported age at onset of PPK ranged from childhood to adulthood. Only 6/22 reported onset in childhood: one before the age of 7 years, two between 7 and 12 years, and three were unable to define the precise age range. Only 6/22 reported the onset of PPK in adolescence (between 13 and 19 years) and 7/22 had PKK in the age of 20 years or above. Three patients were not able to define the age of onset of PPK.

**Table 4:** Dermatological manifestations of patients with the *DSP* c.6310delA p.(Thr2104Glnfs*12) variant.

Focal PPK		21/22 (95%)
Diffuse PPK		1/22 (5%)
**Onset of PPK**	
Childhood (≤ 12 years)		6/22 (27%)
Adolescence (13–19 years)		6/22 (27%)
Adulthood (≥ 20 years)		7/22 (32%)
NA		3/22 (14%)
Wavy or curly hair		22/22 (100%)
Slightly prominent palmoplantar creases		16/22 (73%)
Aquagenic whitening		14/22 (64%)
Palmoplantar hyperhidrosis		13/22 (59%)
Pain in the hyperkeratotic areas		12/22 (55%)
History of or ongoing eczema		9/22 (41%)
Abnormal hair microscopy		3/15 (20%)
Progression of PPK		3/22 (14%)

PPK, palmoplantar keratoderma.

A slight majority (14/22) had observed that hyperkeratosis turned white and spongy after water exposure. Out of 22, 13 reported a hyperhidrosis of the palms and/or soles and three of them a generalized hyperhidrosis. Twelve patients reported pain of the hyperkeratotic areas especially related to fissuring, and 16 patients exhibited slightly prominent palmoplantar creases. Out of 22, 9 had a history of eczema or ongoing eczema. Only 3/22 reported a progression of hyperkeratosis. Notably, all the studied patients had curly or wavy hair, and three of them described their hair as brittle. Light microscopy of hair samples was performed for 15/22, but only three of them had hair shaft abnormalities: one had trichodystrophy, with suspected pili torti, and two had thickened and/or narrowed regions along the hair shafts.

## Discussion

We report nine families with the heterozygous *DSP* c.6310delA p.(Thr2104Glnfs*12) variant associated with PPK, wavy or curly hair, and ACM.

This *DSP* variant was initially identified in a Finn-DCM study in six unrelated Finnish individuals with familial DCM (30). A further analysis of the cardiac phenotype in ten families was conducted by our group (26). This variant was also described in two compound heterozygous patients: both had DCM, woolly hair, fragile skin, and PPK (31, 32). The patient described by Mahoney et al*.* in their study had a Finnish mother, and in the study by Vahlquist et al*.*, the patient was Sweden (31, 32). In gnomAD, the majority of the carriers of this *DSP* variant are of Finnish ethnicity. The allele frequency in the Finnish population is 9.8 times higher than the total allele frequency. As a majority of patients harboring this *DSP* variant are of Finnish ethnicity, we speculate that this variant could represent a Finnish founder variant.

All participants with the *DSP* c.6310delA p.(Thr2104Glnfs*12) variant had PPK and curly or wavy hair. The most common finding was focal PPK focusing on the medial aspect of the first toes, footpad, and heels. It was notable that the degree of hyperkeratosis varied from mild to remarkable thickness even within the same family. In most of the patients in whom the age of onset was defined, hyperkeratosis developed before the age of 20 years and clearly preceded cardiac symptoms.

Similar findings of highly penetrant cutaneous phenotype presenting with PPK or curly hair in ACM patients have been reported (13, 16). In a systematic review, it was found that PPK, combined with hair anomalies and ACM, was associated with variants in desmosomal genes *JUP*, *DSP*, and *DSC2*, and cutaneous features preceded cardiac symptoms (33). Of the other desmosomal genes associated with ACM, curly hair or PPK has been rarely associated with *PKP2* variants (16, 18). In a cohort of patients with *DSP*-related cardiomyopathy, PPK was rarely observed, whereas curly hair was common and more prevalent in those with biventricular cardiomyopathy than in those with RV-or LV-dominant disease (34). Conflicting evidence regarding the relationship between the severity of cutaneous and cardiac features exists (13, 16). As cutaneous features are common and often precede cardiac symptoms in desmosomal ACM, cardiac evaluation is justified in patients with PPK and curly hair.

This *DSP* variant associates with ACM. In cardiac evaluation, some patients fulfilled DCM criteria with a normal RV function, whereas others were primarily diagnosed with ARVC. During follow-up, patients with ARVC presented with biventricular abnormalities. This diverse and changing phenotype has been previously observed in *DSP* cohort studies. Bariani et al*.* observed that, during follow-up, a quarter of their *DSP* cardiomyopathy patients could be classified in a different phenotypic group (34). The intrafamilial phenotypic variation observed in this study as well as in others could indicate the influence of other genetic or environmental factors. The broad phenotypic expression illustrates the significant overlap in the phenotypes of DCM, ARVC, and ACM, which may lead to difficulties in diagnostic and risk stratification (35). The ARVC Task Force criteria has poor sensitivity when diagnosing LV-dominant ACM and *DSP* cardiomyopathy, whereas using the ACM Padua criteria might help increase diagnostic accuracy. (18, 34, 36, 37).

As the participants with the *DSP* variant were relatively young and asymptomatic, we found it unexpected that majority of them had abnormal findings in cardiac evaluation, and thus could be considered affected. Even though most did not fulfill any cardiomyopathy criteria, almost all participants with the *DSP* variant had arrhythmias or abnormalities as revealed by CMR, echocardiography, or laboratory tests. The median age at cardiomyopathy diagnosis was in line with our previous study of the same *DSP* variant; by the age of 60, cardiomyopathy could be diagnosed only in half (26). In other studies, the mean age at diagnosis in *DSP* cardiomyopathy was found to be lower, ranging from 33 to 35 years (18, 34), although, similar to our study, incomplete penetrance and older age at diagnosis were observed in family members (18, 36).

Arrhythmias associated with this *DSP* variant are unpredictable. The increased susceptibility to arrhythmic events manifests as increased VES, NSVT, and sometimes sustained VT or VF. In this study, it was found that in some families, SCDs were prevalent, and sustained ventricular arrhythmias were the first cardiac manifestation rather than ventricular dysfunction. Susceptibility to ventricular arrhythmias was in line with our previous study; almost a third of the patients had VT/VF (26). Patients with pathogenic *DSP* variants are at a high risk of experiencing life-threatening arrhythmias (9, 11, 36). Risk stratification based on ARVC or DCM studies might not be sufficient in patients with *DSP* cardiomyopathy, especially in those with the LV-dominant phenotype. In *DSP* cohort studies, it was found that patients experienced ventricular arrhythmias even with a normal or only mildly reduced systolic function (9, 18, 34). The RV-dominant phenotype is the risk factor and the outcome is the sustained ventricular arrhythmias, (11, 34, 36). More studies are needed to assess the risk of life-threatening arrhythmias in patients with *DSP* cardiomyopathy. The cardiac phenotype and prognosis associated with pathogenic *DSP* variants have been mostly studied with symptomatic patients. It is possible that the less symptomatic family members might have a better prognosis than index patients.

Participants with the *DSP* variant had lower TnI and CRP levels than those with myocarditis. Nine participants with the *DSP* variant had presented at some point with elevated TnI levels without any evident reason. Persistent and fluctuating troponin levels have been observed in *DSP* cardiomyopathies (38). Participants with the *DSP* variant did not demonstrate values outside of the reference range in other inflammatory markers. Laboratory tests were taken in an asymptomatic phase from participants with the *DSP* variant and their family members. It was possible that higher values could be observed during follow-up or during an acute inflammatory episode.

Six participants (21%) with the *DSP* variant had experienced at least one episode of chest pain and elevated troponin levels, resulting in an initial suspicion of viral myocarditis. Other studies have reported patients with pathogenic *DSP* variants presenting with inflammatory episodes. The initial suspicion of acute myocarditis or myocardial infarction has later led to a diagnosis of left-dominant or biventricular ACM (20, 39–42). These episodes have sometimes been associated with exercise (18, 19, 43). Similar to our findings, other studies have found that 15%–20% of *DSP* variant carriers had experienced an episode of myocardial injury presenting with acute chest pain and troponin elevation (18, 34, 36). When the genetic background of patients with myocarditis was evaluated, patients with pathogenic *DSP* variants and family history of ACM were found (40, 44–46). It is unclear if patients harboring pathogenic *DSP* variants have episodes of myocardial injury due to desmosomal disruption that triggers inflammatory response mimicking myocarditis (43). It is also possible that the abnormal myocardial structure predisposes to viral infections that result in myocarditis (44).

CMR, and especially myocardial tissue characterization, is important when assessing patients and family members. In this study, even though echocardiography was unremarkable in relatively asymptomatic family members with the *DSP* variant, a majority of them presented with abnormal findings upon CMR. Volumetrics in CMR or echocardiography did not markedly differ in patients with myocarditis and participants with the *DSP* variant. This could be partly explained by the incomplete penetrance of the DSP variant and the relatively young age of the participants, although normal LV dimensions are not uncommon in the left-dominant ACM (37). All index patients and patients with myocarditis presented with substantial LGE, whereas some family members with the *DSP* variant had none. Participants with the *DSP* variant had a more evenly distributed LGE when compared with patients with myocarditis. A ring-like LGE was observed exclusively in participants with the *DSP* variant. The significance of this pattern is still unclear. It has been suggested to be associated with ACM and sometimes with pathogenic *DSP* variants (21, 47). Non-ischemic ring-like LGE has also been associated with an increased risk of sustained ventricular arrhythmias (48, 49). It can be difficult to differentiate myocarditis from an inflammatory episode of ACM. Similar findings upon CMR were sometimes found in patients with acute myocarditis and participants with the *DSP* variant during a stable phase in this study. In other studies, patients with inflammatory episodes of ACM have been observed to present with edema and subepicardial LGE, as well as volumetric changes in the LV and RV (22). A spotted distribution of LGE could point toward myocarditis (22).

Abnormal trabeculation was exclusively present in participants with the *DSP* variant. Trabeculation was more pronounced in those with the *DSP* variant even when LV size was not markedly increased. Trabeculation has been observed in athletes, healthy volunteers, and patients with other cardiomyopathies, but patients with LVNC have a higher ratio of NC/C myocardium (50). Pathogenic *DSP* variants have been associated with LVNC, and additionally, increased trabeculation fulfilling the LVNC criteria has been observed in patients with *DSP*-associated ACM (3, 9, 35, 46, 51, 52). In addition to the ring-like LGE distribution, increased trabeculation could be a sign of *DSP* cardiomyopathy, but larger datasets are needed to confirm these observations.

## Study limitations

The study population of participants with the *DSP* variant, family members without the *DSP* variant, and patients with myocarditis was small. The participants with clinically diagnosed viral myocarditis did not undergo genetic testing or endomyocardial biopsies during their hospitalization. Echocardiography or CMR findings may not fully reflect all findings related to this *DSP* variant. As the patients were relatively asymptomatic and young, some might develop a more severe cardiac phenotype fulfilling cardiomyopathy criteria in the coming years. Larger datasets are needed to confirm the CMR findings. We have raised the possibility that this *DSP* c.6310delA p.(Thr2104Glnfs*12) variant could represent a Finnish founder variant, but further studies and haplotype analysis are needed to confirm this.

## Conclusions

The *DSP* c.6310delA p.(Thr2104Glnfs*12) variant presents with ACM that may affect the right, left, or both ventricles. Clinical presentation included increased trabeculation in the myocardium and ventricular arrhythmias preceded by PPK and curly or wavy hair.

*DSP*-associated cardiac manifestation can be relatively asymptomatic and difficult to recognize. Intrafamilial variation in phenotype is common. Patients may experience sustained ventricular arrhythmias as a first symptom of cardiomyopathy even with a normal systolic function. A detailed history of arrhythmia symptoms in patients and family members might help identify individuals who need further evaluation with Holter monitoring or even a loop recorder. Dermatologic features already developing in childhood and adolescence might help recognize these patients at an earlier stage. PPK, combined with curly or wavy hair, should trigger cardiac evaluation and a search for the underlying genetic cause.

The possibility of inflammatory episodes related to ACM should be kept in mind when conducting CMR for patients with suspected myocarditis, especially for patients with cutaneous findings, recurrent myocarditis, or a family history of cardiomyopathy or SCD.

## Data Availability

The datasets presented in this article are not readily available because of the need to maintain the privacy of the individuals who participated in the study and to comply with GDPR legislation. Requests to access the datasets should be directed to the corresponding author.
