# Supplementary material for: DSP c.6310delA p.(Thr2104Glnfs*12) associates with arrhythmogenic cardiomyopathy, increased trabeculation, curly hair, and palmoplantar keratoderma
Source: Front Cardiovasc Med. 2023 Mar 15;10:1130903. doi: 10.3389/fcvm.2023.1130903 (PMC10050721; doi:10.3389/fcvm.2023.1130903)
Supplement: Supplementary file 1 [file Datasheet1.docx]

Supplementary Material

# Supplementary Figures and Tables


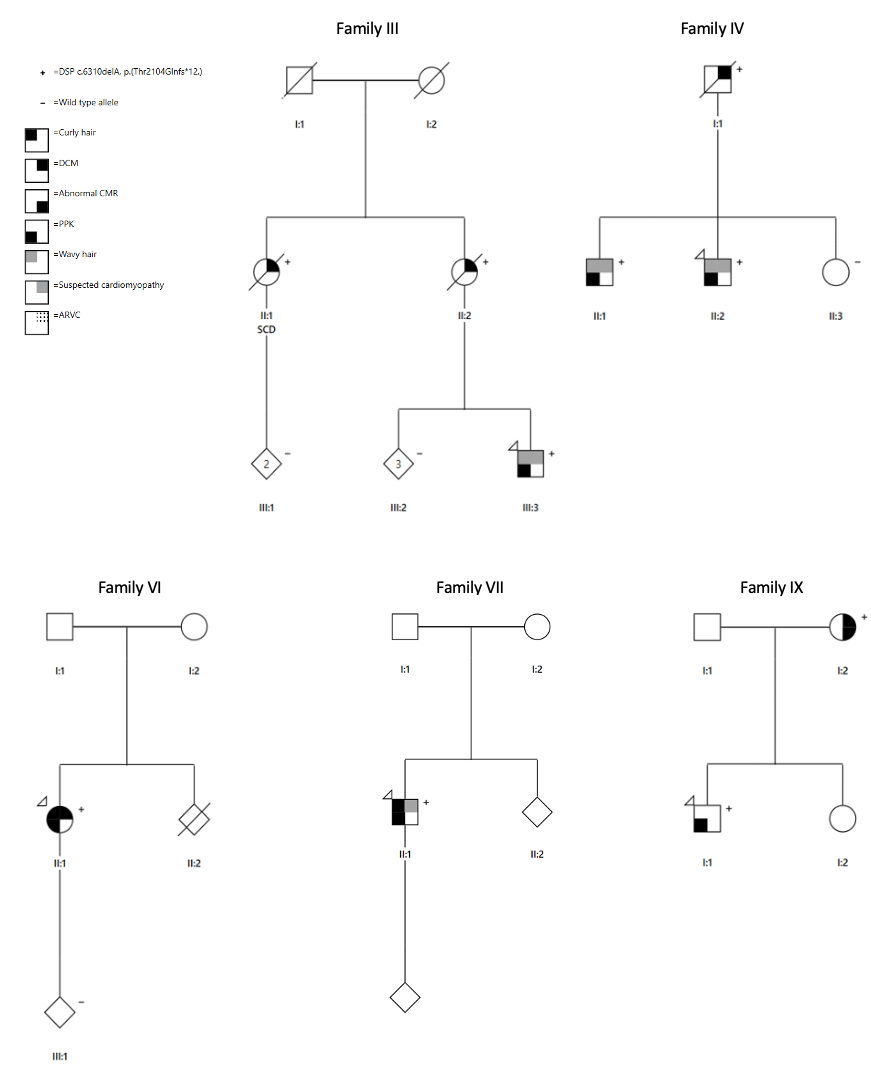


***Supplemental Figure 1.*** Pedigrees of families III, IV, VI, VII, and IX

Arrows indicate the index patients. Genotype: + heterozygous for the *DSP* p.(Thr2104Glnfs*12), - wild type allele. *Right upper quadrant*: black for individual with dilated cardiomyopathy (DCM), dotted for arrhythmogenic right ventricular cardiomyopathy (ARVC), grey for suspected cardiomyopathy. *Right lower quadrant*: black for abnormal cardiac magnetic resonance imaging (CMR). *Left upper quadrant*: black for curly hair, grey for wavy hair. *Left lower quadrant*: black for verified palmoplantar keratoderma (PPK). SCD for sudden cardiac death.
